# Correction: The Covid-19 pandemic in Sweden: Prolonged and unevenly distributed effects on the volume of pediatric anesthesia and surgery demonstrated by data from the Swedish Perioperative Register

**DOI:** 10.1371/journal.pone.0352123

**Published:** 2026-06-18

**Authors:** The PLOS One Staff

S2 Fig is a duplicate of Fig 2 and S3 Fig is a duplicate of Fig 3. Please view the correct S2 Fig and S3 Fig below.

The publisher apologizes for the errors.

## Supporting information

S2 Fig Evolution of waiting time for surgeries on tonsils. (TIF)

S3 Fig Evolution of waiting time for inguinal hernia surgery. (TIF)
