# Supplementary figures and images for: The Covid-19 pandemic in Sweden: Prolonged and unevenly distributed effects on the volume of pediatric anesthesia and surgery demonstrated by data from the Swedish Perioperative Register
Source: PLoS One. 2025 Oct 29;20(10):e0335400. doi: 10.1371/journal.pone.0335400 (PMC12571281; doi:10.1371/journal.pone.0335400)

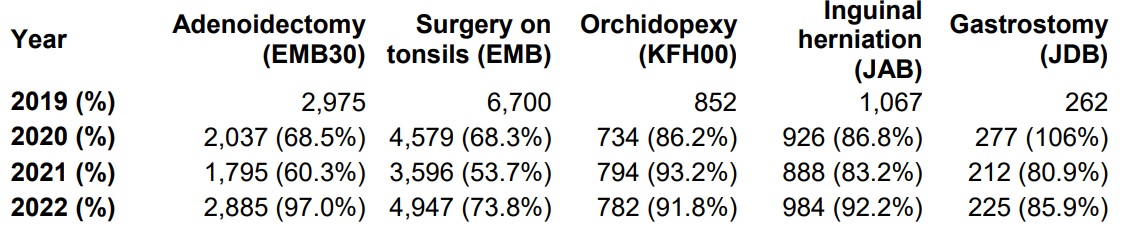

Supplement: S1 Table — Yearly total number and quota compared to 2019 of five selected common pediatric surgeries in Sweden. (JPG) [file pone.0335400.s001.jpg]

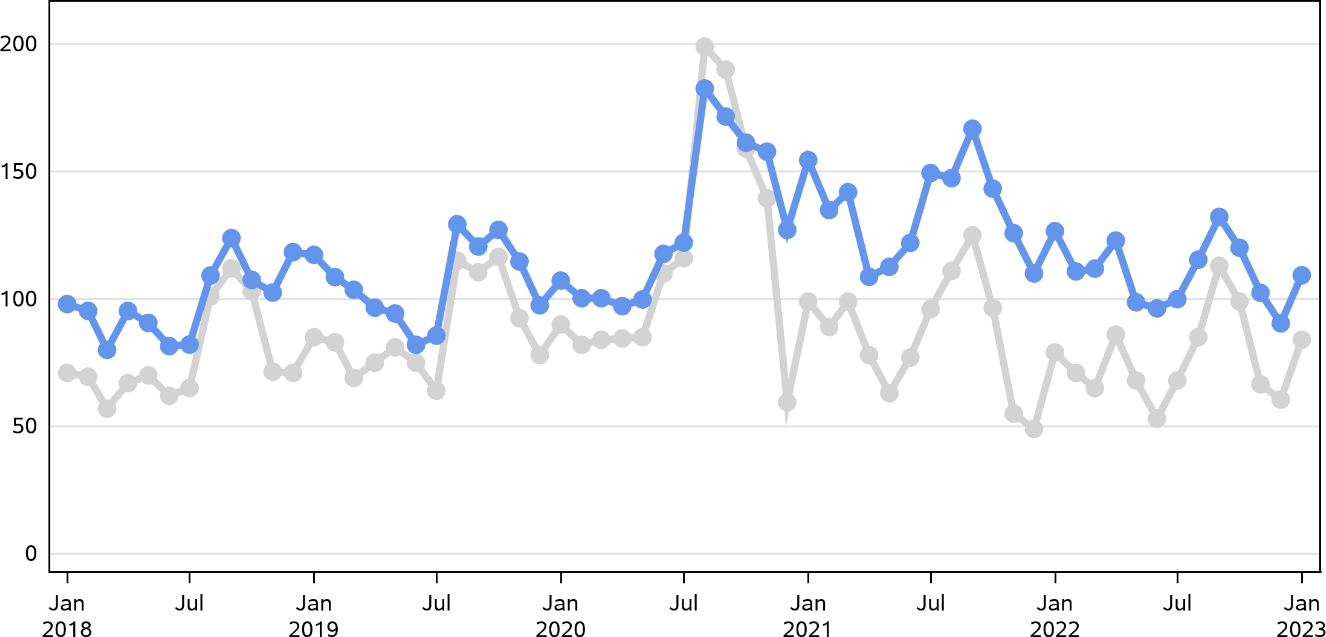

Supplement: S1 Fig — Monthly evolution of waiting time for adenoidectomy (EMB30) shown in days to surgery (blue = mean, gray = median). Waiting time is drawn from data in SPOR by calculating the difference between the time stamp for “Start of surgery” minus the time stamp for “Decision to operate”. (TIF) [file pone.0335400.s002.tif]

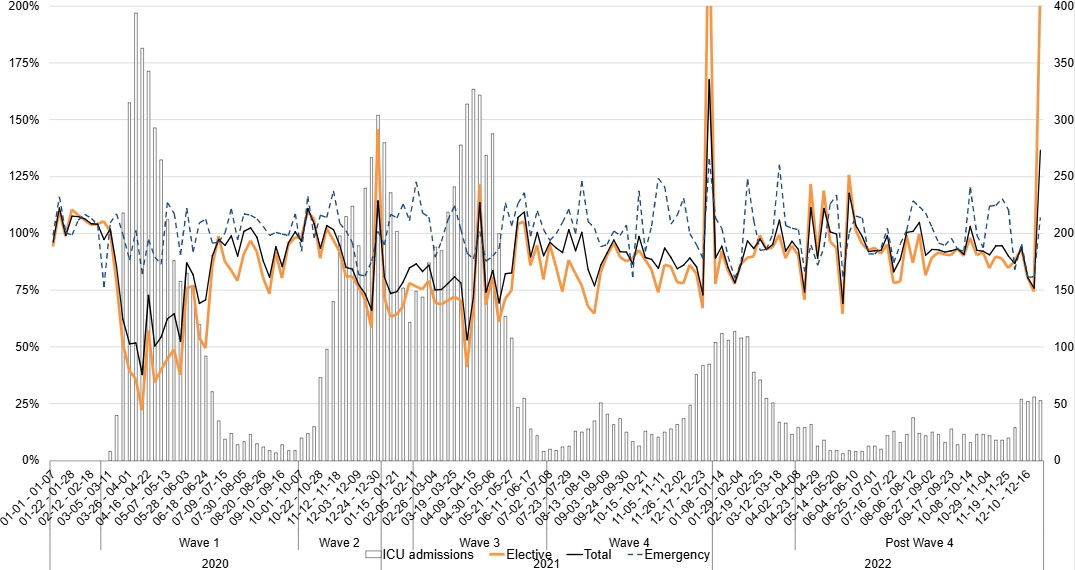

Supplement: S2 Fig — Monthly evolution of waiting time for surgeries on tonsils (EMB) shown in days to surgery (blue = mean, gray = median). Waiting time is drawn from data in SPOR by calculating the difference between the time stamp for “Start of surgery” minus the time stamp for “Decision to operate”. (TIF) [file pone.0335400.s003.tif]

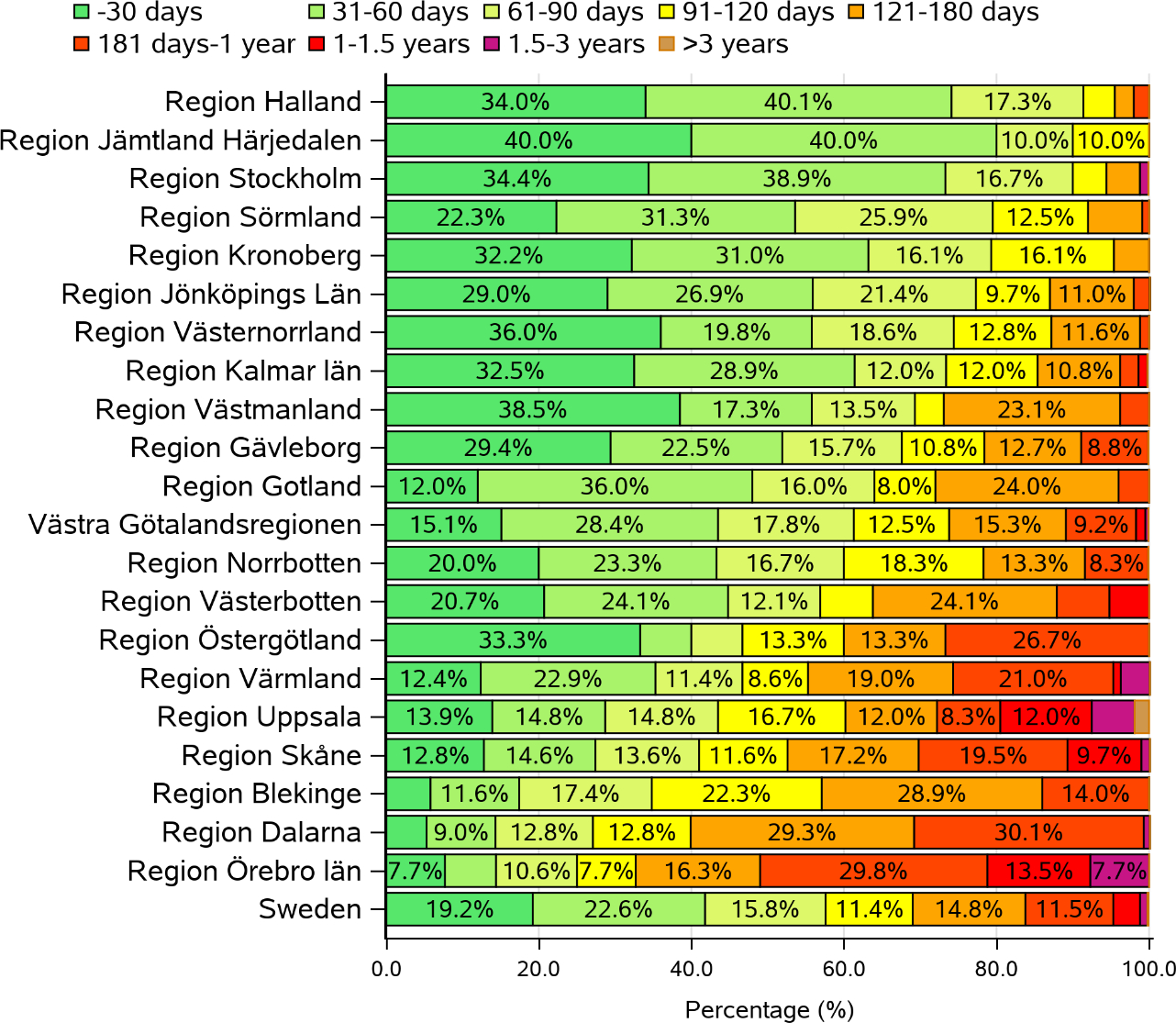

Supplement: S3 Fig — Monthly evolution of waiting time for inguinal hernia surgery (JAB) shown in days to surgery (blue = mean, gray = median). Waiting time is drawn from data in SPOR by calculating the difference between the time stamp for “Start of surgery” minus the time stamp for “Decision to operate”. (TIF) [file pone.0335400.s004.tif]

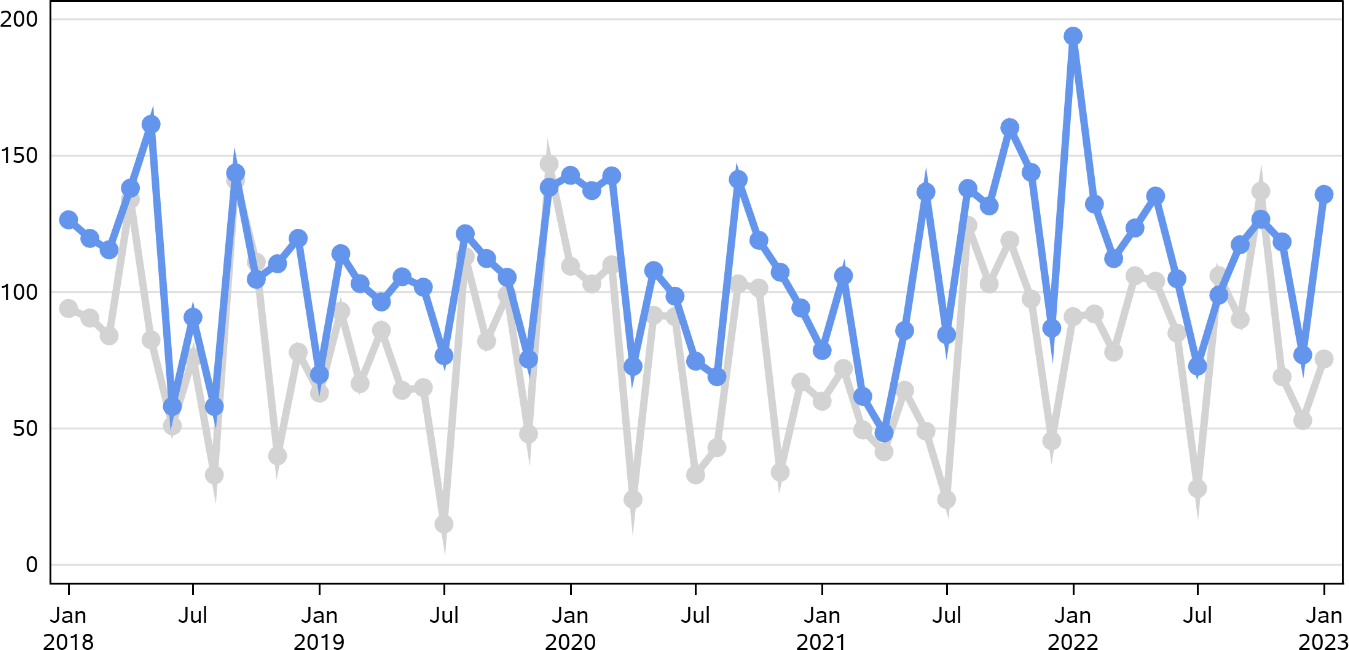

Supplement: S4 Fig — Monthly evolution of waiting time for gastrostomy (JDB) shown in days to surgery (blue = mean, gray = median). Waiting time is drawn from data in SPOR by calculating the difference between the time stamp for “Start of surgery” minus the time stamp for “Decision to operate”. (TIF) [file pone.0335400.s005.tif]

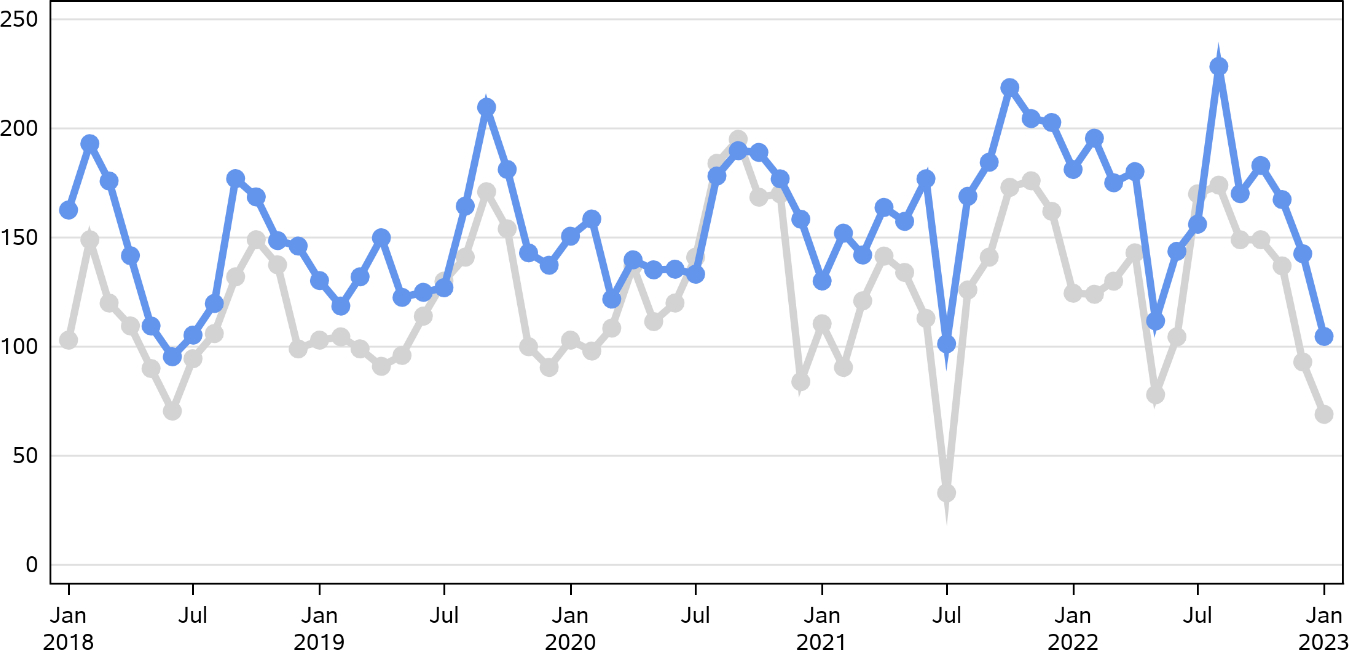

Supplement: S5 Fig — Monthly evolution of waiting time for orchidopexy (KFH) shown in days to surgery (blue = mean, gray = median). Waiting time is drawn from data in SPOR by calculating the difference between the time stamp for “Start of surgery” minus the time stamp for “Decision to operate”. (TIF) [file pone.0335400.s006.tif]
