# Supplementary material for: The Covid-19 pandemic in Sweden: Prolonged and unevenly distributed effects on the volume of pediatric anesthesia and surgery demonstrated by data from the Swedish Perioperative Register
Source: PLoS One. 2025 Oct 29;20(10):e0335400. doi: 10.1371/journal.pone.0335400 (PMC12571281; doi:10.1371/journal.pone.0335400)
Supplement: Attachment — Submitted filename: Response to Reviewers.docx [file pone.0335400.s008.docx]

**Response to Reviewers**

Dear editors,

We thank the reviewer and editors for their feedback on our manuscript. This is a rebuttal letter that aims to respond to the requirements and questions from the journal and the reviewer. We respond to the questions raised one-by-one below, starting with the journal requirements.

Kindly,

Sixten Melander, corresponding author, on behalf of co-authors

Gunnar Enlund, Helene Engstrand Lilja and Peter Frykholm

**Response to journal requirements**

1. Please ensure that your manuscript meets PLOS ONE's style requirements, including those for file naming.

*We have updated the format to meet PLOS ONE style according to requirements specified on the journal website.*

2. Thank you for submitting your work to PLOS ONE. At this time, we require the following information in order to proceed with your submission: As you are reporting a retrospective study, please ensure that you have discussed whether all data were fully anonymized before you accessed them. Please include this information in method statement

*The dataset from SPOR used in this study did not contain complete id information for individual patients. To be precise, we had information on the age at the time of surgery expressed in years and months but not days. Patient name or other identification markers were not available to our research group.*

3. Please update your submission to use the PLOS LaTeX template.

*A LaTeX template will be uploaded with the updated manuscript.*

4. We note that you have indicated that there are restrictions to data sharing for this study. For studies involving human research participant data or other sensitive data, we encourage authors to share de-identified or anonymized data. However, when data cannot be publicly shared for ethical reasons, we allow authors to make their data sets available upon request.

*We have made our data available and updated our Data Availability statement.*

5. Please include captions for your Supporting Information files at the end of your manuscript, and update any in-text citations to match accordingly.

*The supporting information files have had their captions and legends updated to match the publishing guidelines.*

**Response to reviewers**

Reviewer #1: Thank you for providing the opportunity to review this paper, entitled "Swedish pediatric surgery during Covid: A national registry study on the effects of the pandemic on pediatric anesthesia and surgery ". Below, I have provided my evaluation and comments.

1. I request information about the data source (SPOR) in the method section. How is data recorded into the register? Is it done manually? Are SPOR validated? Most important, to what extent does SPOR cover all operations performed in Sweden? Do centers from both the public and private sectors report to SPOR? Tonsillectomy is often performed in private centers, and sometimes surgery is offered outside the patient's region. Could this impact the validity of the results?

*Thank you for your questions regarding SPOR. We have tried to elaborate and clarify your questions on the register both in the manuscript and below.*

How is data recorded and validated?

*The methods of reporting and validation are specified in Ref 5. For your convenience we have provided the abstract of Ref 5:*

*Background: Since 2013 surgical units in Sweden have reported procedures to the national Swedish Perioperative Register (SPOR). More than four million cases have been documented. Data consist of patient ID, type of surgery, diagnoses, time stamps during the perioperative process (from the decision to operate to the time of dis-charge from the postoperative recovery area) and quality measures. This article aims to describe SPOR and validate data mapping. Also, we wished to illustrate the utility of the SPOR in assessing variations in national surgical capacity during the COVID-19 pandemia years 2020–2021.*

*Methods: After a detailed description of SPOR, we report on the validation of data performed by comparing data from local databases with data stored in the central SPOR database, assessing missing values and accuracy. Effects of the pandemic on surgical capacity were described by developing an index, based on the number of performed surgical procedures per week during four production weeks in January2020. Subsequent weeks were then compared with this baseline.*

*Results: The validation effort demonstrated nearly 100% data accuracy for the number and type of surgical procedures between local and central data. Missing data was a problem for some parameters. The number of performed surgical procedures decreased dramatically from week 11 in 2020 compared with normal production on a national basis, mainly impairing elective surgery.*

*Discussion: Data validation revealed good agreement between local and central data-bases. The changes in national surgical capacity during the pandemic were illustrated by an index based on the reported surgical production.*

*Holmström B, Enlund G, Spetz P, Frostell C. The Swedish Perioperative Register: Description, validation of data mapping and utility. Acta Anaesthesiol Scand. 2023 Feb;67(2):233-239. doi: 10.1111/aas.14174. Epub 2022 Dec 14. PMID: 36424870; PMCID: PMC10108284.*

*Regarding if data is manually reported, the answer is no. The perioperative process has been defined in SPOR as the period from the decision to operate until the operation has been performed and the patient is discharged from postoperative care. The process occurs in four phases: operation notification, planning, surgery and postoperative care. Each phase includes several well-defined variables, some of which are mandatory. The variables are grouped according to these four phases. Certain basic data about each patient (e.g., date of birth, Swedish personal identity number or coordination number, administrative gender) are mandatory. The variables consist of patient data, time stamps, codes for the surgical procedure and diagnoses. Outcomes and quality parameters (e.g., perioperative bleeding, patient body temperature, postoperative pain, nausea/vomiting and selected vital parameters) are also reported. Every night, surgical units report their activity—consisting of all surgical interventions performed during the last 24 h with these variables—to the SPOR. The variable list contains parameters that can be used to assess quality and production. With daily reporting from all publicly run hospitals, SPOR offers a timely and detailed overview of Sweden's surgical production.*

Does SPOR cover all operations performed in Sweden?

*Sweden has a strong tax-funded healthcare available for all. Private clinics are very rare, and the few that exist have small caseloads compared to the publicly funded hospital system. There are no dedicated private paediatric hospitals in Sweden, and the largest ambulatory clinic performs 800 GA-circumcisions yearly in boys ≥ 3 y (the clinic's age limit). Some private hospitals with a few pediatric procedures register data in the Swedish Perioperative Register (included in the category small hospital) while a few office-based ENT clinics do not. Today SPOR covers 100 % of operations performed in government-run hospitals in Sweden. SPOR receives data regarding about 40 % of the operations done in the private sector; they are included is the present paper as small hospitals. The private hospitals that report to SPOR are mainly orthopedic and general surgery. No private ENT-surgery has been reported yet. In Sweden the amount of ENT-surgery that is done in the private sector relatively limited so in our assessment it does not impact the validity of our results. We estimate that presently less than 2000 procedures with GA are performed annually in Swedish private clinics not connected to SPOR, which would amount to less than 1% missing data for this reason. A note has been added to the limitations section.*

2. The title contains "…anesthesia and surgery." However, the results do not focus on anesthesia. Are all surgical interventions included in the results performed under anesthesia or specifically general anesthesia? This needs to be clarified.

*Thank you for this comment. We agree that the focus of the paper is more of the effects of the pandemic on surgical specialties than specific anesthesia-related caseload although anesthesia services were affected simultaneously. The register includes all procedures performed with anesthesia services involved, e.g sedation for oncologic or radiologic diagnostic procedures besides general anesthetics.* *We have clarified this in the methods section and changed the title.*

3. Only centers that had started reporting to SPOR before 2019 were included. Were all these centers reporting to SPOR throughout the entire period from 2019 to 2022? Or have some centers stopped reporting to the register due to increased workload during the pandemic? This needs to be clarified.

*All hospitals connected to SPOR upload their data automatically to the register monthly. The uploading process has been validated for all data fields used in this study (*see reference in question 1)*. We decided early in the planning of the study to only include centers that reported data all four years. We did identify two small hospitals with a total caseload of < 1000 cases per year that stopped their reporting after 2021 and were therefore excluded. We have no information if they stopped reporting due to the pandemic per se or because of increased centralization or re-organization of patient flow during these years. An additional 7 hospitals were excluded since they joined SPOR after 2019.*

4. I also request information about the variables retrieved from SPOR and the data arrangement. For example, I find it hard to understand the calculation of the evolution of waiting times. This needs to be clarified in the manuscript.

*SPOR has invented a new variable called “The real waiting time.”*

*For every operation that is completed, SPOR automatically calculates the difference between the time stamp for “Start of surgery” minus the time stamp for “Decision to operate”. In that way SPOR gets the real waiting time expressed as mean and median for each type of surgery. Due to that we can follow how the waiting time changes over time.*

5. The large volume of data analyzed means that even small differences can be statistically significant (which could be seen in the analysis of mean age at surgery). Therefore, the results need to be interpreted from the perspective of what is clinically relevant. However, under the statistical section, the significance level that was considered significant is missing.

*A P-value of less than 0,05 was considered significant. This has been clarified in the stastical methods section.*

6. Several data points appear to be wrong in the tables. For example, the data in Table 1, column 2019, row total-elective, appears to be incorrect (4,067?). The data in Table 3, column Tonsil surgery, row 2019, also seems wrong (1.6?). Please check all data presented in the manuscript.

*Thank you for spotting these errors due to formatting within Microsoft Excel which automatically removed endings with zeros in them from the correct numbers (40,670 and 6,700) to 4,067 and 6,7 respectively. This was not intended and has now been corrected.*

7. The discussion is weak and needs to be revised. Some results are briefly discussed, and some statements are poorly substantiated. For instance, the text suggests that ENT surgeries, including tonsillectomy, can be postponed for months or even years without serious harm. The two main indications for tonsil surgery are upper airway obstruction, causing abnormal ventilation during sleep, and infection-related problems such as recurrent tonsillitis. Studies and national guidelines argue that these patients should be prioritized due to short- and long-term consequences of postponed surgery. The discussion section should acknowledge the potential harm of postponed surgeries.

*We have updated our discussion section to make it more thorough. Specifically, we have added more information regarding the potential dangers of post-poning tonsillectomies and grommets, and why surgical planning for ENT-procedures specifically were difficult as well as the risks of post-poning surgery for orchidopexy.*
