# Supplementary material for: The Covid-19 pandemic in Sweden: Prolonged and unevenly distributed effects on the volume of pediatric anesthesia and surgery demonstrated by data from the Swedish Perioperative Register
Source: PLoS One. 2025 Oct 29;20(10):e0335400. doi: 10.1371/journal.pone.0335400 (PMC12571281; doi:10.1371/journal.pone.0335400)
Supplement: Attachment — Submitted filename: Response_to_Reviewers_auresp_2.docx [file pone.0335400.s009.docx]

**Response to Reviewers**

Dear editors and reviewers,

We thank the reviewers for their feedback on our manuscript. This is a rebuttal letter that aims to respond to the questions from the reviewers. We respond to the questions raised one-by-one below.

Kindly,

Sixten Melander, corresponding author, on behalf of co-authors

Gunnar Enlund, Helene Engstrand Lilja and Peter Frykholm

Reviewer #2: This manuscript addresses an important and timely topic, contributing to our understanding of long-term healthcare implications post-COVID-19 on healthcare systems. It is a commendable effort to analyze data from a national cohort.
However, significant revisions are necessary to enhance the methodological rigor and ensure the claims are well-supported by the presented data. Specifically, the conclusions regarding long-term effects of COVID-19 require a more cautious interpretation, given the descriptive nature of the retrospective cohort study and potential biases in the analysis. Furthermore, a more robust time series analysis and careful control for confounding factors are critical to substantiate the findings. I do not agree with the concluding claim that the provided data is evidence for long-term effects of COVID-19, at least based on the evidence provided so far. We observe data indicating an increasing overall stress burden on healthcare worldwide due to various reason that also include i.e. changing demographics, rising costs etc.. These factors are and probably cannot addressed in a cohort study based on retrospective secondary data.

*Thank you for this comprehensive review with constructive comments and helpful suggestions. We have revised the conclusion and have added a discussion in the limitations on the problems of cause and effect and confounding. Obviously, we cannot provide the complete picture in this retrospective study. We have also added a rudimentary time series analysis by the addition of data from the official four waves of the pandemic.*

**Introduction**
1) Include more references; ensure all claims are supported by citations.

*We have provided new references, marked in red.*

**Methods**
1) Report the methods and study characteristics more thoroughly.

*We have expanded the methods section as suggested.*

2) Explain the rationale for selecting 2019 as the baseline year. Please adjust the research question and objectives accordingly. Elaborate on why comparisons were not made to an average of the years before.

*The reason for using only 2019 as the baseline year was that this was the first year in which almost all Swedish Hospitals had joined the Registry. Ideally, we would have had access to complete baseline data from at least three years but since the Register was still lacking data from several major centers, inclusion of data reported earlier than 2019 would have been less reliable.*

3) Provide details of the study setting, including the exact number of study centers and the variables used for secondary analyses (e.g., level of care).

*We have provided the number of hospitals in each category contributing with data to SPOR.*

4) Include comprehensive details of the cohort, including eligibility criteria and the sources and methods of participant selection.

*SPOR is national registry with the aim of including all Swedish hospitals with anesthesia and surgery services. All hospitals connected to SPOR were therefore eligible for inclusion in the present study. We received a complete dataset from SPOR for the studied period and excluded only those 9 hospitals that had not reported data from the complete study period. This was mentioned in the limitations. We decided from the start to exclude children <16 years old, and they were not in our initial data. A cohort flow chart has been added for clarification.*

5) Clearly define all outcomes, exposures, predictors, potential confounders, and effect modifiers.

*We have tried to more clearly define outcomes and confounders in the methods section.*

6) Expand on data sources and measurement methods, including a discussion of the registry as a secondary data source and its likely purpose for billing.

*We have added some text to further describe SPOR.*

7) Describe efforts to address potential sources of bias in the registry or clarify if this is a convenience sample.

*We have added the information of convenience sample in statistical methods.*

8) Explain how quantitative variables were handled in the analyses. If applicable, describe groupings and provide a rationale for the chosen groupings (e.g., why focus on the 20 most common procedures in 2019 rather than overall procedures?).

*Quantitative variables have been clearly described and the rationale for groupings has been expanded in the methods. We did focus on reduction in overall procedures as the primary outcome. The choice of more detailed analysis of the 20 most common procedures was arbitrary, but was included to attempt to provide more detail for common procedures. It would not have been feasible to report data at this level for each and every type of procedure, as the total number of different procedure codes was 3,276, most of them with low total numbers of registered entries.*

9) Provide the rationale for investigating the mean age at the time of surgery and how it relates to the study objectives.

*The purpose of the study was to investigate patterns and possible effects of the reduced capacity during the pandemic. Mean age at surgery for a specific procedure could be a marker for postponing specific elective operations. Since data on when a procedure is planned and performed were included in the extracted dataset, we also decided to look at waiting times.*

10) Include more robust time-series analyses, such as interrupted time-series analysis (Especially given the claim in the conclusion).

*Regarding time series analysis, Figure 1 describes a time series analysis with weekly changes in case-load against the backdrop of ICU admissions as a measure of the intensity of the pandemic. We have added data on changes during the pandemic waves as a rudimentary intervention time series analysis.*

10.1) Consider the influence of different COVID-19 waves, comparing them to baseline and different timings ((i.e. <https://doi.org/10.1177/014107682096244>, <https://doi.org/10.1186/s12889-023-15375-7>, <https://doi.org/10.1371/journal.pgph.0000029>).

*Thank you for this suggestion. We have added information on changes during the waves in Fig 2 and Table 2.*

10.2) Consider causal impact analysis (<https://doi.org/10.2147/rmhp.s459307>).

*It was not in the scope of the present study include a causal impact analysis since it would have required additional data.*

13) Explain how missing data were handled in the study.

*The SPOR has an excellent automated data collection system with extensive validation that provides complete datasets for the variables we analysed. SPOR provides several other interesting variables such as adverse events (including grade) that are less well validated and therefore prone to varying amounts of missing data. This is why we focused on core data such as date, age, and type of procedure.*

14) Provide the r source code for the analysis.
*This is the source code:*

library(openxlsx)

infile1 <- "K:/Academy/UU/Frykholm/Indata/Tabell 1 HT23_utan_losen.xlsx"

adb <- read.xlsx(infile1)

orig <- adb

colnames(adb)[3:6] <- paste0("X", colnames(adb)[3:6])

colnames(adb)[c(9,11,13)] <- c("p1", "p2", "p3")

adb$X2020 <- as.numeric(gsub(" |\\(.*", "", adb$X2020))

adb$X2021 <- as.numeric(gsub(" |\\(.*", "", adb$X2021))

adb$X2022 <- as.numeric(gsub(" |\\(.*", "", adb$X2022))

adb$X2022 <- as.numeric(gsub(" |\\(.*", "", adb$X2022))

colnames(adb)[12] <- "19_22"

adb[,c("20_21", "p4", "20_22", "p5", "21_22", "p6")] <- NA

for(i in 1:17){

  adb[i,"19_20"] <- poisson.test(c(adb$X2019[i], adb$X2020[i]),c(adb$X2019[18],adb$X2020[18]))$estimate

  adb[i,"p1"] <- poisson.test(c(adb$X2019[i], adb$X2020[i]),c(adb$X2019[18],adb$X2020[18]))$p.value

  adb[i,"19_21"] <- poisson.test(c(adb$X2019[i], adb$X2021[i]),c(adb$X2019[18],adb$X2021[18]))$estimate

  adb[i,"p2"] <- poisson.test(c(adb$X2019[i], adb$X2021[i]),c(adb$X2019[18],adb$X2021[18]))$p.value

  adb[i,"19_22"] <- poisson.test(c(adb$X2019[i], adb$X2022[i]),c(adb$X2019[18],adb$X2022[18]))$estimate

  adb[i,"p3"] <- poisson.test(c(adb$X2019[i], adb$X2022[i]),c(adb$X2019[18],adb$X2022[18]))$p.value

  adb[i,"20_21"] <- poisson.test(c(adb$X2020[i], adb$X2021[i]),c(adb$X2020[18],adb$X2021[18]))$estimate

  adb[i,"p4"] <- poisson.test(c(adb$X2020[i], adb$X2021[i]),c(adb$X2020[18],adb$X2021[18]))$p.value

  adb[i,"20_22"] <- poisson.test(c(adb$X2020[i], adb$X2022[i]),c(adb$X2020[18],adb$X2022[18]))$estimate

  adb[i,"p5"] <- poisson.test(c(+adb$X2020[i], adb$X2022[i]),c(adb$X2020[18],adb$X2022[18]))$p.value

  adb[i,"21_22"] <- poisson.test(c(adb$X2021[i], adb$X2022[i]),c(adb$X2021[18],adb$X2022[18]))$estimate

  adb[i,"p6"] <- poisson.test(c(adb$X2021[i], adb$X2022[i]),c(adb$X2021[18],adb$X2022[18]))$p.value

}

write.xlsx(adb, "K:/Academy/UU/Frykholm/Poisson_2023-11-09.xlsx")

**Results**
15) Clarify whether cases were excluded and provide details about the overall initial sample. Report the number of individuals at each stage of the study (e.g., eligible, examined for eligibility, confirmed eligible, included).

*This information has been added.*

16) If possible, include a cohort flow chart detailing exclusions and other relevant stages.

*Thank you for this suggestion. A flow-chart has been added.*

17) Ensure all tables in the PDF are fully included to allow for a complete evaluation of the results.

*The tables have been reformatted*

18) Align the results with the research question (hypothesis) and objectives. For instance, results such as age characteristics do not fit the stated focus. Adjust the methods and introduction if the scope is broader than the stated patterns of reduction.

*We have expanded our methods section, please see answer below for clarification.*

18.1) In particular state the rationale behind studying the age. It is not stated in the introduction, methods or discussion what it hast to do with patterns in the reduction of pediatric procedures or if it is a cofactor effecting reduction. The effect of age is not statistically addressed.

*We hypothesized that massive postponement of minor surgery could lead to children having their surgery at an older age. This issue is explored in table 3. We think confidence intervals is an appropriate way to report this kind of data rather than a long list of p-values.*

19) no infferential results are presented, aside from the statement in the methods section.
*This is not a classic epidemiological study. We had access to data on procedures and the age and sex of the patients but no data on confounders such as socioeconomic factors. We did however use poisson regression to compare the pandemic years with the preceding year regarding the number of performed procedures in relation to the population during the respective years.*

**Discussion**
19) Provide a cautious overall interpretation of the results and discuss their generalizability.

*We have revised the interpretation and conclusion.*

20) Address the effect of comparing one pre-COVID year to multiple post-COVID years.

*Added this discussion to the limitations.*

21) Discussion evaluation is challenging due to missing results (e.g., incomplete tables).

*We apologize for this inconvenience. Apparently the tables were truncated at page margins. This has been addressed in the current version.*

22) Clarify the claim that "the strength of the present study is that it provides detailed data from a national perioperative database including almost all major Swedish hospitals with pediatric anesthesia." Provide evidence to support this claim and elaborate on what "almost all major" entails, as well as its implications for representativeness.

*Sweden has a tax-funded healthcare system and the vast majority of pediatric surgery is performed in government-run hospitals which are 100% covered in SPOR. A few small private clinics perform office-based procedures in children without reporting their data to SPOR. We estimate that less than 2000 procedures with GA are performed annually in Swedish private clinics, which would amount to less than 1% missing data for this reason. We have added info to the limitations section.*

23) Discuss the study's limitations in more detail, particularly regarding the chosen methods. Consider potential sources of bias or imprecision, addressing both their direction and magnitude.

*The limitations section was expanded.*

24) Please relate to other studys that study the impact of covid on admission and surgeries.

*We have expanded our discussion on the topic of admissions and volume of surgery and added four more references.*

**Overall**
24) Reduce the number of figures in the appendix to only those that are directly relevant, I was overwhelmed

*To overwhelm was not our intention. We included these figures to provide detailed regional data but detailed comparative analysis was not within the scope of the present paper.*

25) I cannot fully evaluate the manuscript, as not all data are included (tables do not fit the manuscript).

*Tables have been reformatted.*
