# Supplementary figures and images for: Correction: The Covid-19 pandemic in Sweden: Prolonged and unevenly distributed effects on the volume of pediatric anesthesia and surgery demonstrated by data from the Swedish Perioperative Register
Source: PLoS One. 2026 Jun 18;21(6):e0352123. doi: 10.1371/journal.pone.0352123 (PMC13278423; doi:10.1371/journal.pone.0352123)

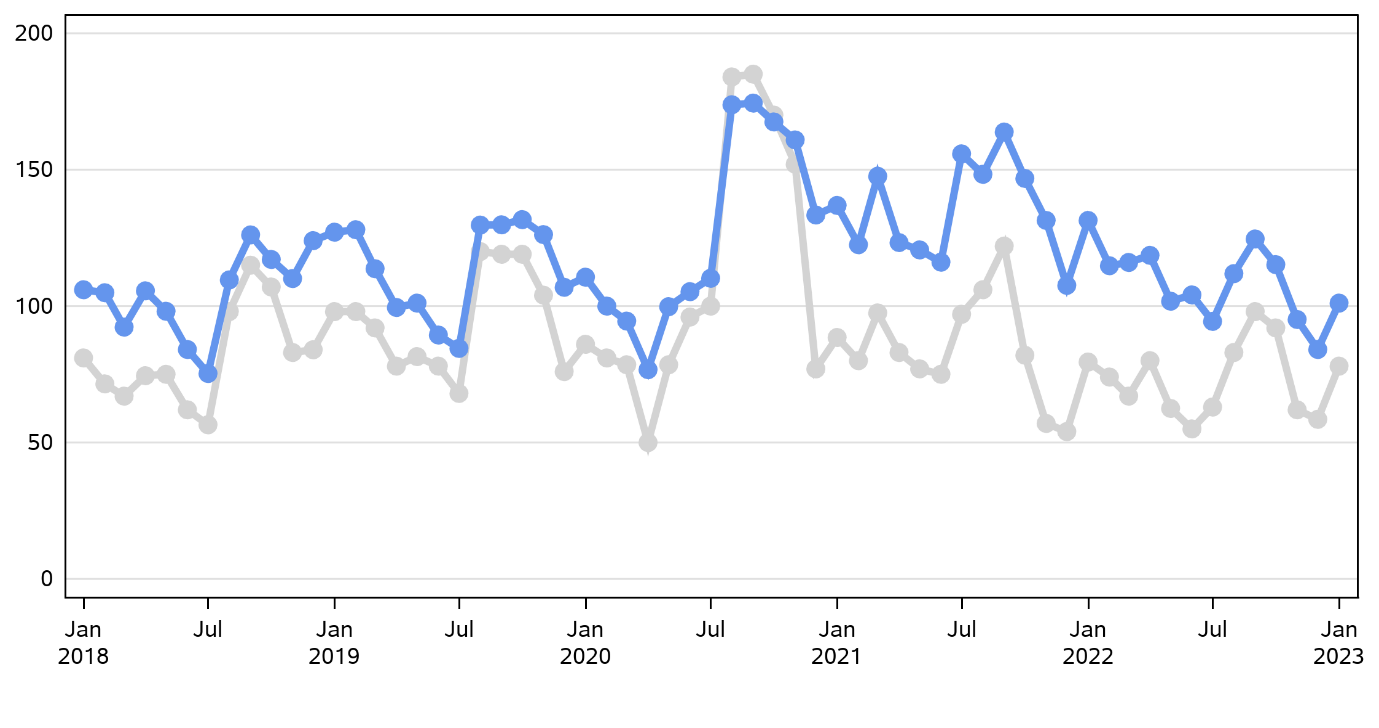

Supplement: S2 Fig — (TIF) [file pone.0352123.s001.tif]

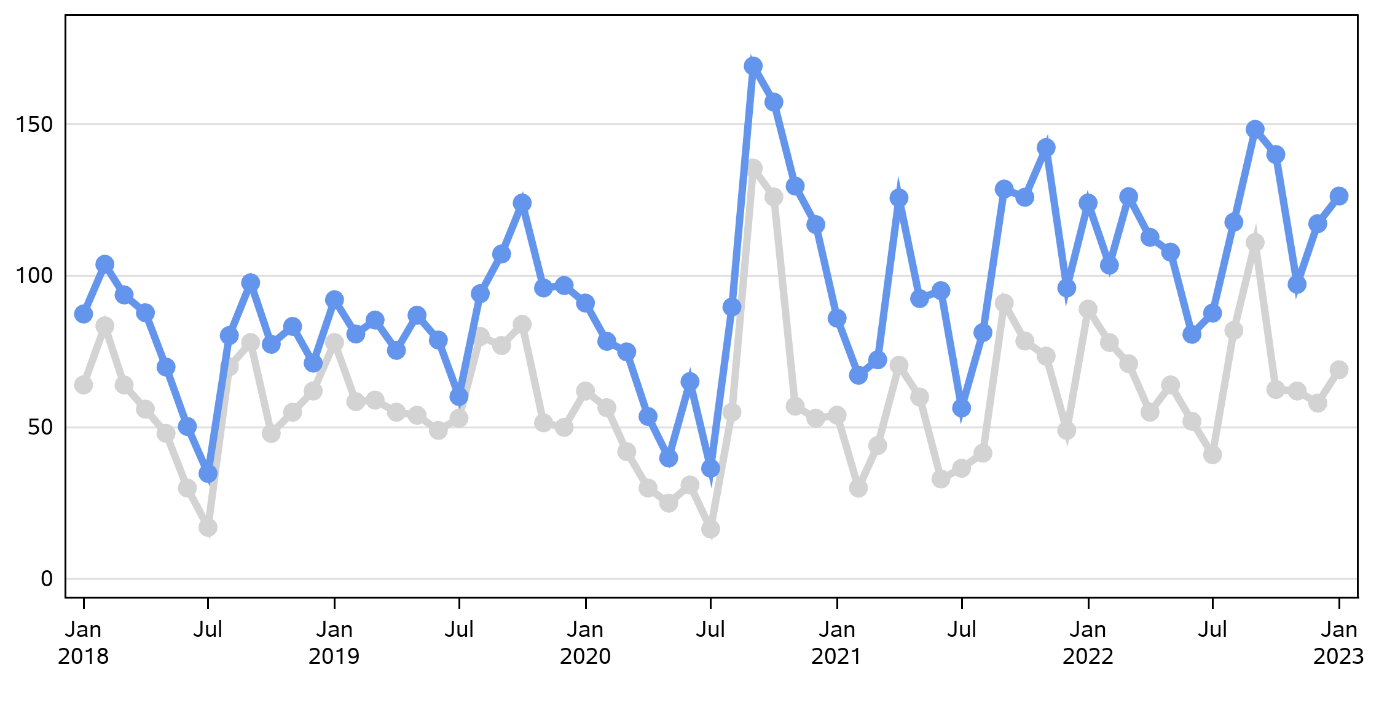

Supplement: S3 Fig — (TIF) [file pone.0352123.s002.tif]
